# Cigarette smoke‐induced urothelial cell damage: potential role of platelet‐activating factor

**DOI:** 10.14814/phy2.13177

**Published:** 2017-03-07

**Authors:** Shannon E. Kispert, John Marentette, E. Cristian Campian, T. Scott Isbell, Hannah Kuenzel, Jane McHowat

**Affiliations:** ^1^ Department of PathologySaint Louis University School of MedicineSaint LouisMissouri; ^2^ Department of Obstetrics, Gynecology & Women's HealthSaint Louis University School of MedicineSaint LouisMissouri

**Keywords:** Inflammation, interstitial cystitis, smoking, urothelium

## Abstract

Cigarette smoking is an environmental risk factor associated with a variety of pathologies including cardiovascular disease, inflammation, and cancer development. Interstitial cystitis/bladder pain syndrome (IC/BPS) is a chronic inflammatory bladder disease with multiple etiological contributors and risk factors associated with its development, including cigarette smoking. Previously, we determined that cigarette smoking was associated with bladder wall accumulation of platelet activating factor (PAF), a potent inflammatory mediator that facilitates transendothelial cell migration of inflammatory cells from the circulation. PAF has been shown to reduce expression of tight junctional proteins which could ultimately lead to increased urothelial cell permeability. In this study, we observed that cigarette smoke extract (CSE) treatment of human urothelial cells increases PAF production and PAF receptor expression and reduces wound healing ability. After exposure to cigarette smoke for 6 months, wild‐type C57BL/6 mice displayed urothelial thinning and destruction which was not detected in iPLA_2_ *β* ^−/−^ (enzyme responsible for PAF production) animals. We also detected increased urinary PAF concentration in IC/BPS patients when compared to controls, with an even greater increase in urinary PAF concentration in smokers with IC/BPS. These data indicate that cigarette smoking is associated with urothelial cell damage that may be a result of increased PAF‐PAF receptor interaction. Inhibition of iPLA_2_ *β* activity or blocking of the PAF‐PAF receptor interaction could serve as a potential therapeutic target for managing cigarette smoke‐induced bladder damage.

## Introduction

A sedentary lifestyle, overeating, excessive alcohol consumption, and smoking can contribute directly to many diseases, including diabetes, cardiovascular disease, cancer, and inflammatory diseases (Verheij et al. 1981a; McHowat et al. 1993a; Sharma et al. 2009). Bladder conditions such as lower urinary tract symptoms (LUTS), urinary tract infections (UTI), interstitial cystitis/bladder pain syndrome (IC/BPS), and bladder cancer have been shown to be influenced by lifestyle choices (Park et al. 1994). IC/BPS is a chronic inflammatory bladder disease with significant morbidity (Siebeck et al. 1994; Messner et al. 2008). IC/BPS has multiple etiological contributors such as mast cell infiltration, neurogenic involvement and impairment of urothelial integrity (Yamada et al. 1994; Sharma et al. 2010). Impairment of the normal barrier properties of the urothelium can result in leakage of urine contents into the bladder wall, resulting in pain and inflammation (Yamada et al. 1994; Sharma et al. 2014). Smoking is a modifiable risk factor associated with IC/BPS (Wu et al. 1993; Yan et al. 1993) and has been shown to contribute to exacerbation of IC/BPS symptoms (McHowat et al. 1993b). According to the Interstitial Cystitis Association, IC/BPS patients who smoke have noted symptom improvement upon smoking cessation.

We have previously shown that exposure of human bladder microvascular endothelial cells (HMVEC‐Bd) to cigarette smoke extract (CSE) resulted in the production of platelet activating factor (PAF) and increased inflammatory cell adherence to the endothelial cell surface. This observation could be reduced by pretreatment of HMVEC‐Bd with *(S)*‐bromoenol lactone (BEL), an inhibitor of calcium independent phospholipase A_2_ *β* (iPLA_2_ *β*) activity, which is necessary for PAF production (Kasckow et al. 1986; McHowat et al. 2001; Jenkins et al. 2002). Additionally, we observed increased inflammatory cell recruitment in the bladder wall of wild‐type mice exposed to cigarette smoke with a significant reduction in inflammatory cell aggregates in the bladder wall of iPLA_2_ *β* knockout (iPLA_2_ *β* ^−/−^) mice exposed to cigarette smoke (McHowat et al. 1993a). Using immortalized urothelial cells, we observed a significant redistribution of iPLA_2_ isoforms, with a significant increase in iPLA_2_ *β* immunoprotein and activity in cells isolated from IC/BPS patients when compared to controls (Singer et al. 2002). The increased iPLA_2_ *β* activity in IC/BPS‐derived urothelial cells resulted in the increased PAF production and polymorphonuclear leukocyte (PMN) adherence in response to tryptase stimulation (Singer et al. 2002).

Our previous studies suggest that cigarette smoking can directly contribute to bladder inflammation through increased PAF production. In addition to its role in inflammatory cell recruitment (Park et al. 1994; McHowat et al. 2001), PAF has been shown to be involved in processes that ultimately damage urothelial cell integrity such as increasing expression and activity of matrix metalloproteinases (MMP) (Burke and Dennis 2009; Dennis et al. 2011) which can disrupt epithelial integrity. In an in vitro model of inflammatory bowel disease (IBD) using Caco‐2 cells, it was shown that administration of PAF reduces the expression of tight junction proteins and reduces transepithelial electrical resistance (Xu et al. 2012). We hypothesize the cigarette smoke‐induced PAF production could impact urothelial cell integrity and contribute to symptoms in IC/BPS patients.

## Materials and Methods

### Cell culture

Primary human urothelial cells (HUC) were obtained from ScienCell Research Laboratories (Carlsbad, CA). Immortalized urothelial cells were derived from cell isolations from bladders of normal (four donors) or IC/BPS (four donors) patients via bladder washing during cytoscopy. All patients were never smokers. Cells were immortalized with a retrovirus encoding the oncoproteins E6 and E7 of human papillomavirus type 16 and selected for stable integration of the retroviral pro virus with G418. Urothelial cell cultures were grown in EpiLife Media (Cascade Biologics, Inc. Portland, OR) with calcium (0.06 mmol/L), growth factor supplements provided by the manufacturer and penicillin (20 U/mL)/streptomycin (100 mg/mL) (Sigma Chemical Company, St. Louis, MO) and incubated at 37°C, with an atmosphere of 95% O_2,_ 5% CO_2_. Confluent monolayers were differentiated by adding 1 mmol/L calcium and 10% fetal bovine serum (Ca/FBS). Experiments were performed after 3 days of differentiation. Cells were treated with cigarette smoke extract (CSE, 20 *μ*g/mL) for indicated times as previously described (McHowat et al. 1997). CSE was obtained from Murty Pharmaceuticals (Lexington, KY). Urothelial cells used in these studies have been previously demonstrated to form fully differentiated stratified epithelial culture with thin, tightly opposed apical superficial cells and more loosely connected underlying cells (Stafforini et al. 2003; Beckett and McHowat 2008) and stained positively for cytokeratin expression (Beckett and McHowat 2008).

### Measurement of iPLA_2_ activity

Urothelial cell cultures were washed with ice‐cold PBS and suspended in 500 *μ*L PLA_2_ assay buffer containing (mmol/L): sucrose 250, KCl 10, imidazole 10, EDTA 5, dithiothreitol 2, with 10% glycerol, pH = 7.8. The suspension was sonicated and centrifuged to remove cellular debris, nuclei, and mitochondria. The supernatant was centrifuged at 100,000*g* for 60 min to separate cytosol and membrane fractions. The pellet was resuspended in PLA_2_ assay buffer and activity was assessed by incubating enzyme (8 *μ*g protein) with 100 *μ*mol/L (16:0, [^3^H]18:1) plasmenylcholine substrate in buffer containing (mmol/L): Tris 10, EGTA 4, 10% glycerol, pH = 7.0 at 37°C for 5 min in a total volume of 100 *μ*L butanol. Released radiolabeled fatty acid was isolated by application of 25 *μ* of the butanol phase to channeled Silica Gel G plates, development in petroleum ether/diethyl ether/acetic acid (70/30/1, v/v/v) and subsequent quantification by liquid scintillation spectrometry.

### Measurement of PAF production

Human urothelial cells grown to confluence were incubated with Hanks' balanced salt solution containing 10 *μ*Ci of [^3^H] acetic acid for 20 min at room temperature. After experimental conditions, cell lipids were extracted using the Bligh and Dyer method (Verheij et al. 1981b). Total lipid extracts were resuspended in 9:1 CHCl_3_:MeOH and applied to TLC plates. Plates were developed in 100:50:16:8 chloroform, methanol, acetic acid, and water. The region corresponding to PAF was scraped and measured by liquid scintillation counting.

### Measurement of urinary PAF

Human urine samples were collected from female patients in accordance with a Saint Louis University Institutional Review Board approved protocol from control (*n* = 6), IC/BPS nonsmokers (*n* = 6) and IC/BPS smokers (*n* = 6). Urine collection was obtained by normal void at the time of office visit. Three 10 mL aliquots were immediately frozen in liquid nitrogen and stored at −80°C until analysis. Urinary PAF concentration was measured directly using an ELISA kit (Biotang, Waltham, MA). Urinary aliquots (100 *μ*L) were added to microtiter plates with a biotin‐conjugated polyclonal antibody specific for PAF. PAF content in samples was determined spectrophotometrically at 450 nm using a Synergy 2 microplate reader (Biotek, Winooski, VT). PAF content was normalized to urinary creatinine levels.

### Measurement of urinary creatinine concentration

Previously frozen human urine specimens collected for PAF measurement were thawed at 4C, mixed, and centrifuged. A 500 *μ*L aliquot was used for creatinine assay. Urine creatinine was measured on an Abbott Architect chemistry analyzer using the following enzymatic method. In the first reaction creatinine is hydrolyzed to creatine by the enzyme creatininase. The creatine is subsequently hydrolyzed to sarcosine and urea by the same enzyme creatininase. The sarcosine is then oxidized to glycine and formaldehyde with the concomitant production of hydrogen peroxide by the enzyme sarcosine oxidase. The hydrogen peroxide formed in turn reacts with 4‐aminoantipyrine and *N*‐ethyl‐*N*‐sulfopropyl‐*m*‐toluidine (ESPMT) in the presence of a peroxidase to yield the colored product quinoneimine dye which can be measured spectrophotometrically at 548 nm. The change in absorbance at 548 nm is proportional to the creatinine concentration in the sample.

### Measurement of urothelial cell wound healing

Urothelial cell growth was determined using the electric cell‐substrate impedance sensing (ECIS) system (Applied Biophysics, Troy, NY) as described previously (Fleer et al. 1981). Cells were grown on ECIS electrode arrays (8W1E). The impedance fluctuations of cell attachment and spread were continuously monitored over 24 h. An alternating current of 1 *μ*A at 4 kHz was applied between a small sensing electrode (250*‐μ*m diameter) and a large counter electrode. Once a monolayer had formed, calcium chloride (1 mmol/L) and fetal bovine serum (10%) were added to the growth media to induce differentiation. Once a stable impedance was achieved for 24 h, CSE (20* μ*g/mL) was added to the culture medium. Wounding of cells was achieved using a 6V signal at 45 kHz for 30 sec. Application of this field results in a rapid drop in impedance of cell layers due to the death of the cells on the electrode. Impedance increases as cells migrate from the perimeter of the electrode to replace the wounded cells.

### Immunoblot analysis

Urothelial cells were suspended in lysis buffer containing (mmol/L) HEPES 20 (pH 7.6), sucrose 250, dithiothreitol 2, EDTA 2, EGTA 2, *β*‐glycerophosphate 10, sodium orthovanadate 1, phenylmethylsulfonyl fluoride 2, leupeptin 20 *μ*g/mL, aprotinin 10 *μ*g/mL, and pepstatin A 5 *μ*g/mL. Cells were sonicated on ice and centrifuged at 20,000*g* at 4°C for 20 min to remove cellular debris and nuclei. Cytosolic protein was separated by SDS/PAGE and electrophoretically transferred to nitrocellulose membranes (Bio‐Rad, Richmond, CA). The blocked nitrocellulose membrane was incubated with primary antibody (anti‐PAF receptor, 1 in 1000 dilution, Cayman Chemical Co., Ann Arbor, MI) and horseradish peroxidase‐conjugated secondary antibody (anti‐rabbit, 1 in 10,000 dilution, Fisher Scientific). Regions of antibody‐binding were detected using enhanced chemiluminescence (Amersham, Arlington Heights, IL) after exposure to film (Hyperfilm, Amersham). Equal loading was verified by immunoblot analysis for *β*‐actin.

### In vivo cigarette smoke exposure

6‐month‐old, female C57BL/6 and iPLA_2_ *β* ^−/−^ mice were exposed to cigarette smoke generated from the University of Kentucky 3R4F research cigarette with the SciReq InExpose system (Montreal, QC, Canada) using the Federal Trade Commission/International Standard Organization standard of 35‐mL puffs of 2 sec duration taken once a minute. Mice were exposed to cigarette smoke for 48 min/day, 5 days/week for 6 months. Experiments were performed according to a Saint Louis University Institutional Review Board approved protocol.

### Mouse bladder histology

Mouse bladders were fixed in 10 % buffered formalin, embedded in paraffin, and cut into 5 *μ*mol/L thick sections. Tissue was deparaffinized and rehydrated in xylene and decreasing concentrations of reagent alcohol. Slides were stained with filtered Gills III hematoxylin (Harleco) and blued in saturated lithium carbonate solution. Slides were immersed in Eosin‐Y Alcoholic (Richard‐Allan Scientific), dehydrated, coverslipped, and viewed under a light microscope.

### Immunohistochemistry

Bladders for immunohistochemistry were removed from animals and fixed in 10 % buffered formalin, embedded in paraffin, and cut into 5 *μ*m thick sections. Tissue was deparaffinized and rehydrated in xylene and decreasing concentrations of reagent alcohol. Sections underwent heat‐induced antigen retrieval using a citric acid‐based antigen unmasking solution (Vector Labs). Sections were incubated with blocking buffer and primary antibody (PAF (1:50 Abbiotec, San Diego, CA), PAF receptor (1:50 Cayman Chemical Co., Ann Arbor, MI), overnight. Sections were washed and incubated with anti–rabbit secondary antibody. Immunohistochemistry was completed with use of the Vectastain Elite Universal ABC system and DAB (Biogenex). Slides were counterstained with filtered Gills III hematoxylin and blued in saturated lithium carbonate solution and viewed under a light microscope.

### Statistical Analysis

All studies were repeated at least in triplicate. Data were analyzed using ANOVA. Differences were regarded as significant at *P* < 0.05 and highly significant at *P* < 0.01. Data are means ± SEM.

## Results

### CSE exposure increases PAF production in human urothelial cells

**Figure 1:**
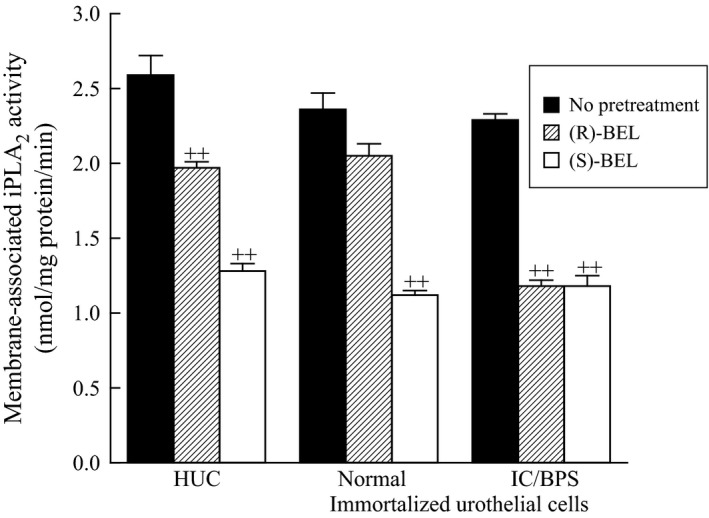
Membrane‐associated calcium‐independent phospholipase A_2_ activity (iPLA_2_) in human urothelial cells (HUC), and in immortalized urothelial cells isolated from control patients (normal) and patients with interstitial cystitis/bladder pain syndrome (IC/BPS). Membrane fractions were incubated in the absence (black bars) or presence of 5 *μ*mol/L bromoenol lactone (BEL) to inhibit iPLA_2_ activity ((*S*)‐BEL to inhibit iPLA_2_ *β*, open bars, or (*R*)‐BEL to inhibit iPLA_2_ *γ*, hatched bars). iPLA_2_ activity was measured in the absence of calcium (1 *μ*mol/L EGTA) using 100 *μ*mol/L (16:0, [^3^H]18:1) plasmenylcholine substrate. Data shown represent the mean + SEM for four separate membrane preparations. ++*P* < 0.01 when comparing activity in the presence or absence of BEL, one‐way ANOVA, Dunnett's test for posthoc comparisons. BPS, bladder pain syndrome.

In previous studies, we have determined that PAF production is mediated by activation of iPLA_2_ *β* (Singer et al. 2002; Sharma et al. 2010). We measured iPLA_2_ activity in human urothelial cells (HUC) and immortalized urothelial cells from control bladders (normal) and IC/BPS patients (IC/BPS) in the absence of calcium and using (16:0, [^3^H]18:1) plasmenylcholine (Fig. 1). iPLA_2_ activity measured in the presence of (*R*)‐bromoenol lactone ((*R*)‐BEL, 5 *μ*mol/L) to inhibit iPLA_2_ *γ* resulted in approximately 50% inhibition of iPLA_2_ activity (Fig. 1, hatched bars) in all cells tested. Incubation with (*S*)‐BEL (5 *μ*mol/L) to inhibit iPLA_2_ *β* resulted in approximately 20% inhibition of activity in HUC, but no significant inhibition of activity in normal cells (Fig. 1, open). These data indicate that the majority of iPLA_2_ activity in human urothelial cells or immortalized urothelial cells from bladders of subjects without IC/BPS is contributed by iPLA_2_ *γ*. In contrast, iPLA_2_ activity in urothelial cells isolated from IC/BPS patients was inhibited by 50% in the presence of (S)‐BEL (Fig. 1, hatched bars), indicating an increase in iPLA_2_ *β* activity in IC/BPS‐derived cells when compared to normal.

**Figure 2:**
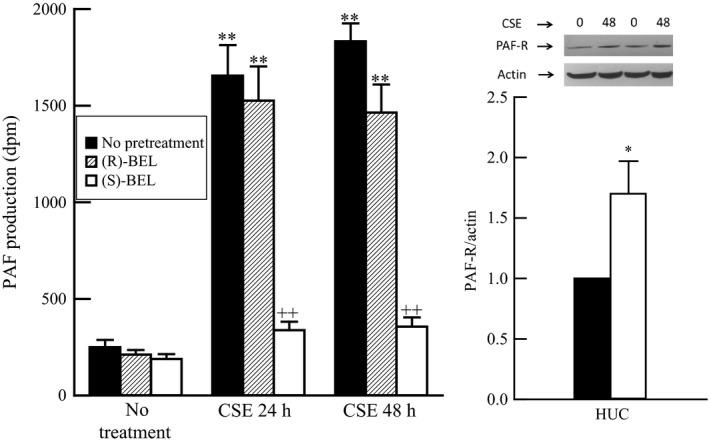
Left panel: Increase in platelet‐activating factor (PAF) content in human urothelial cells exposed to cigarette smoke extract (CSE, 20 *μ*g/mL) for 24 or 48 h (black bars). Increased PAF content is inhibited by pretreatment with (S)‐bromoenol lactone ((*S*)‐BEL, 5 *μ*mol/L, 20 min prior to CSE addition, open bars) to inhibit iPLA_2_ *β*, but largely unaffected by pretreatment with (R)‐BEL (5 *μ*mol/L, 20 min prior to CSE addition, hatched bars) to inhibit iPLA_2_ *γ*. **P < 0.01 when comparing PAF content in the presence or absence of CSE. ++*P *< 0.01 when comparing PAF content in the presence or absence of BEL, one‐way ANOVA, Dunnett's test for posthoc comparisons. Data shown represent the mean + SEM for three separate cell cultures. Right panel: Increased PAF receptor expression in human urothelial cells exposed to CSE (20 *μ*g/mL, 48 h, open bars) when compared to medium alone (black bars). *P < 0.05 when comparing expression in the presence or absence of CSE. Data shown represent the mean + SEM for four separate cell cultures.

To determine the effect of CSE on urothelial PAF production, HUC were incubated with CSE (20 *μ*g/mL) for 24 and 48 h. A significant increase in PAF production was observed in HUC incubated with CSE (Fig. 2, left panel, black bars) when compared to cells incubated in medium alone. Pretreatment of HUC with the iPLA_2_ *γ* specific inhibitor, (*R*)‐BEL (5 *μ*mol/L, 20 min prior to CSE addition) resulted in minimal effect on CSE‐induced PAF production (Fig. 2, left panel, hatched bars). However, pretreatment with the iPLA_2_ *β* specific inhibitor, (*S*)‐BEL (5 *μ*mol/L, 20 min prior to CSE addition), significantly inhibited CSE‐induced PAF production (Fig. 2, left panel, open bars). From these data, we conclude that the predominant isoform of iPLA_2_ responsible for the generation of PAF in HUC is iPLA_2_ *β*. In addition to increased PAF production, we observed a significant increase in PAF receptor expression in HUC exposed to CSE for 48 h (Fig. 2, right panel).

**Figure 3:**
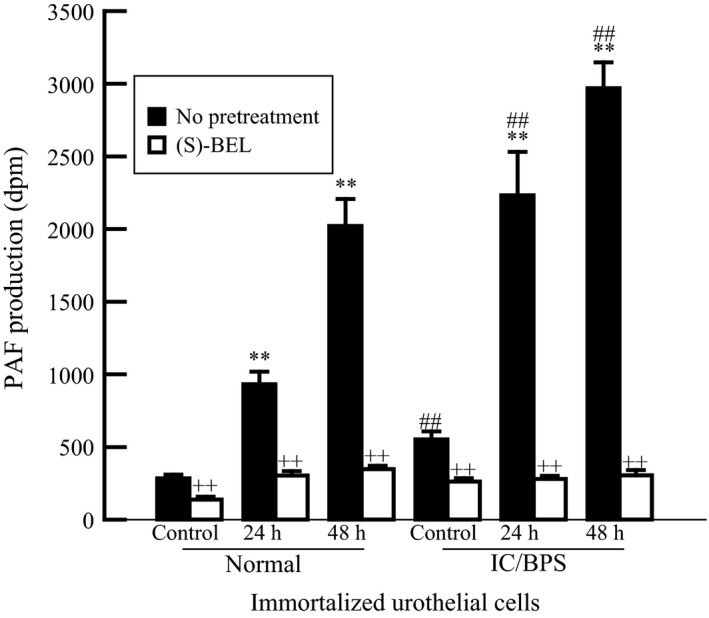
PAF content in immortalized urothelial cells isolated from control bladders (normal) and interstitial cystitis bladders (IC/BPS) exposed to CSE (20 *μ*g/mL) for 24 or 48 h. Increased PAF content is inhibited by pretreatment with (*S*)‐BEL (5 *μ*mol/L, 20 min prior to CSE addition). **P < 0.01 when compared to control. ++*P* < 0.01 when comparing values in the presence (open bars) or absence (black bars) of *(S)*‐BEL. ##*P* < 0.01 when comparing corresponding values between normal and IC/BPS samples, one‐way ANOVA, Dunnett's test for posthoc comparisons. Data shown represent the mean ± SEM for three separate cell cultures from three separate patients in each group. BPS, bladder pain syndrome.

Data obtained in Figures 1 and 2 indicate that iPLA_2_ *β* activity is primarily responsible for PAF production and that iPLA_2_ *β* activity is enhanced in urothelial cells isolated from IC/BPS bladders when compared to normal bladders. Thus, we propose that PAF production would be enhanced in IC/BPS‐derived urothelial cells. To explore this hypothesis we exposed immortalized urothelial cells from IC/BPS patients and control patients to CSE and measured PAF production (Fig. 3). Consistent with data obtained using HUC, we observed a significant increase in PAF production when urothelial cells isolated from non‐IC/BPS patients were incubated with CSE (Fig. 3, Normal, black bars). However, the increase in the IC/BPS patient‐derived urothelial cells were substantially higher than that observed in immortalized normal urothelial cells (Fig. 3, IC/BPS). In addition, IC/BPS patient‐derived urothelial cells in medium produce more basal PAF than in control urothelial cells (Fig. 3). The increase in PAF production was blocked by pretreatment with the iPLA_2_ *β* inhibitor, *(S)*‐BEL, in both cell groups (Fig. 3, open bars).

### PAF concentration in urine

**Figure 4:**
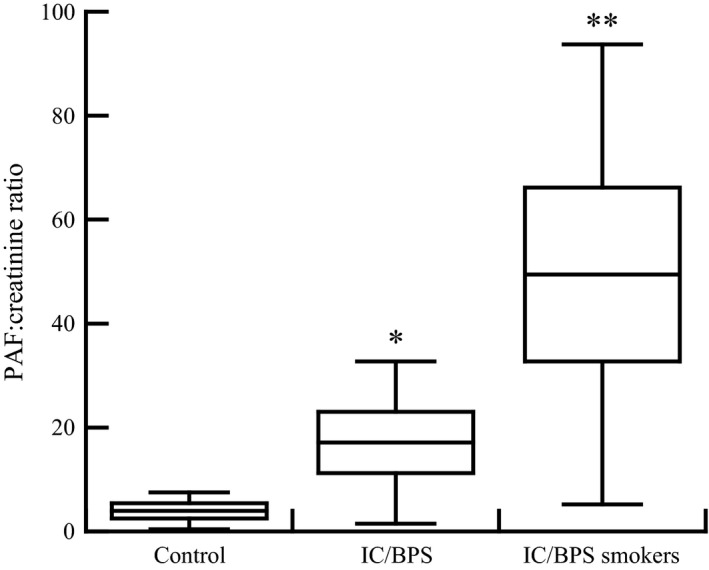
Urine PAF content normalized to creatinine in patients scheduled for cystoscopy with hydrodistention. Urine was collected from non IC/BPS patients (control), IC/BPS patients who are nonsmokers (IC/BPS) and IC/BPS who are current smokers (IC/BPS smokers). *P < 0.05. **P < 0.01 when comparing PAF content in IC/BPS patients to controls, one‐way ANOVA, Dunnett's test for posthoc comparisons. Data shown represent the mean ± SEM for six patients in each group. BPS, bladder pain syndrome.

Urine samples were collected from IC/BPS and non‐IC/BPS patients scheduled for cystoscopy with hydrodistention. Urinary PAF concentrations were measured and normalized to creatinine content. Urine PAF content was significantly greater in IC/BPS patients compared to non‐IC/BPS controls (Fig. 4). Urinary PAF content was significantly higher in IC/BPS patients who smoked than in IC/BPS patients who were nonsmokers (Fig. 4). These data suggest smoking could exacerbate the inflammatory response in IC/BPS patients via bladder PAF content.

### CSE exposure reduces urothelial cell wound healing rate

**Figure 5:**
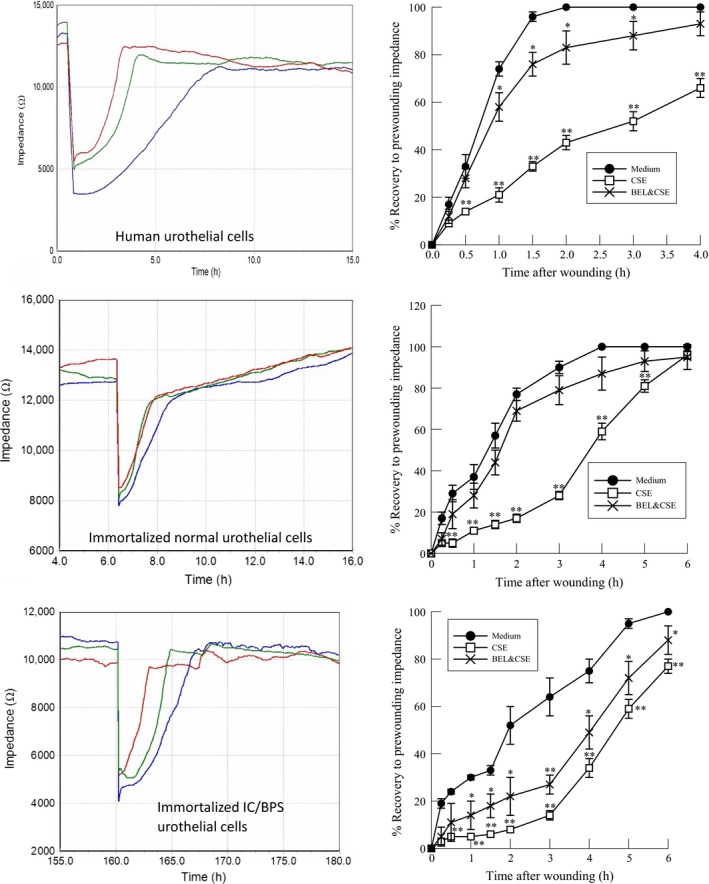
Rate of wound healing in human urothelial cells in the presence of CSE (20 *μ*g/mL, left panels blue lines) or in medium alone (left panels, red lines). Pretreatment with (*S*)‐BEL (5 *μ*mol/L, 20 min prior to CSE addition, left panels, green lines) increased rate of wound healing when compared to CSE alone. Top: Human urothelial cells. Middle: Immortalized urothelial cells isolated from non‐IC/BPS bladders. Bottom: Immortalized urothelial cells isolated from IC/BPS patients. Left panels: Representative data of real time impedance measurements. Right panels: Mean ± SEM impedance values for six separate cell cultures. *P <0.05, **P < 0.01 when comparing values in the presence or absence of CSE. BPS, bladder pain syndrome; CSE, cigarette smoke extract.

Following urothelial cell damage and impairment of the barrier function, it is imperative to reestablish an intact and continuous urothelium to limit the exposure of the bladder wall to potentially irritating urinary components. Previous studies have shown reduced wound healing in cigarette smokers versus nonsmokers (Balsinde and Dennis 1996b; Six and Dennis 2000). To determine the effect of CSE on the wound healing ability of urothelial cells, cells were grown on gold electrodes and impedance measured in real time. Once stable impedance was detected, a high current was applied, killing the cells growing on the active electrode while leaving the surrounding cells intact (Wegener et al. 2002; Wiesner et al. 2008). Wounding and associated cell death are indicated by the sharp drop in impedance observed in Figure 5 (left panel). Impedance increases as cells migrate from the perimeter of the electrode inward to replace the wounded cells (Fig. 5, left panels). Figure 5 shows impedance measured in HUC (top panels), immortalized urothelial cells isolated from control bladders (middle panels) and immortalized urothelial cells from IC/BPS patient bladders. In all three cell types used, we observed a significant delay in wound healing in the presence of CSE (left panels, blue lines) when compared to healing rates of cells in medium alone (left panels, red lines). Wound healing rates were lower in IC/BPS urothelial cells than normal cells in both the presence and absence of CSE (Fig. 5). When cells were pretreated with (*S*)‐BEL (5 *μ*mol/L, 20 min prior to CSE, left panels, green lines) wound healing rates in all cells was significantly greater than in the presence of CSE alone (Fig. 5). These data provide evidence that cigarette exposure directly affects the ability of urothelial cells to reepithelialize a wound following injury suggesting that an IC/BPS patient who is also a smoker may experience similar difficulty in healing the damaged urothelium. In addition, pretreatment with (*S*)‐BEL prior to CSE addition resulted in increased wound healing rates when compared to CSE alone, indicating that targeting PAF accumulation in the bladder wall may be a therapeutic target to manage IC/BPS.

### In vivo cigarette smoke exposure results in disruption of urothelial integrity in mice

**Figure 6:**
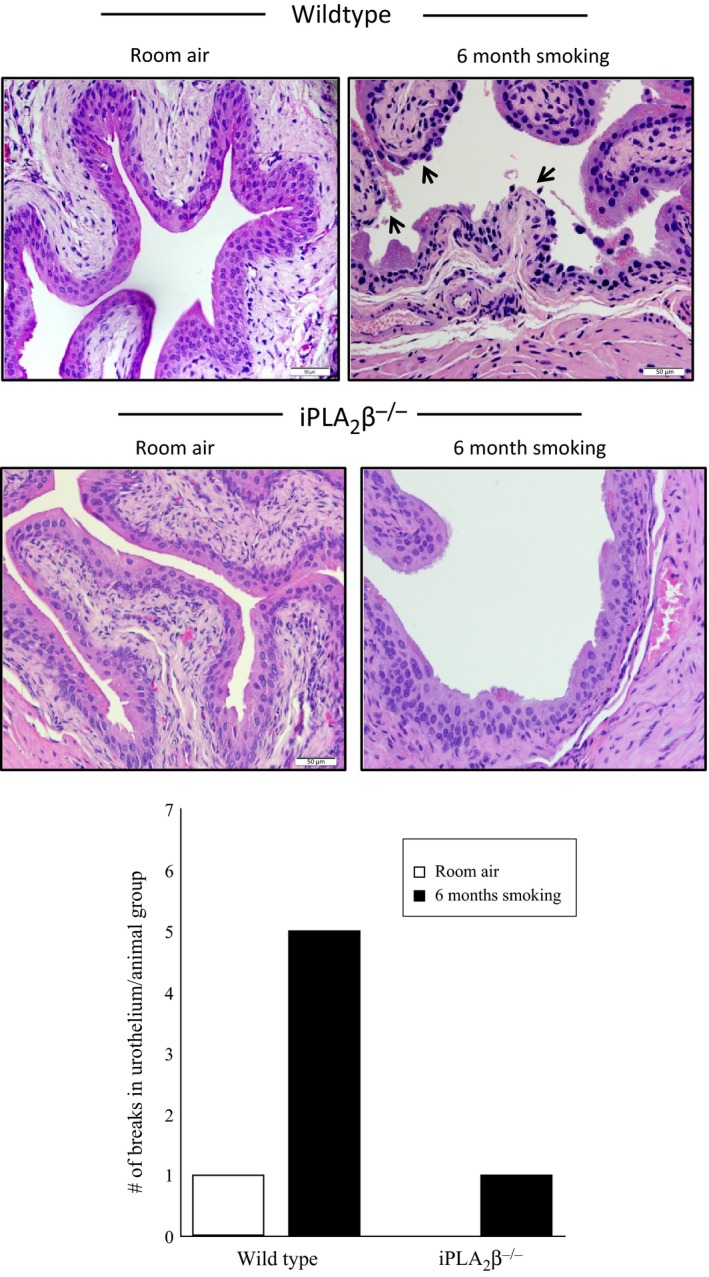
Representative hematoxylin and eosin‐stained sections of mouse bladder. Top panels: Bladder sections from wild‐type female mice exposed to room air (left) or 6 months cigarette smoke (right). Breaks in the urothelium are indicated by the arrows. Middle panels: Bladder sections from iPLA_2_ *β* ^−/−^ female mice exposed to room air (left) or 6 months cigarette smoke (right). Bottom panel: Number of urothelial breaks detected in each animal group, normalized to 1.0 for the wild‐type mice exposed to room air. Six animals in each group.

To determine the impact of smoking on urothelial integrity in vivo we exposed female C57BL/6 mice to mainstream cigarette smoke for 6 months. In wild‐type mice exposed to room air, we observed normal and uniform urothelial thickness (Fig. 6, upper panel). However, in wild‐type mice exposed to cigarette smoke for 6 months, we detected areas of urothelial thinning and denudation (Fig. 6, upper panel, arrows) which would impact the urothelial barrier properties in mice exposed to cigarette smoking.

Data in Figures 1 and 2 show that iPLA_2_ *β* is the predominant isoform responsible for PAF production in urothelial cells. To support the potential role of PAF may be playing in inducing damage to urothelial cell integrity in response to cigarette smoke exposure, we administered cigarette smoke to iPLA_2_ *β* ^−/−^ mice in the same manner as their wild‐type counterparts. As shown in Figure 6 middle panels, the iPLA_2_ *β* ^−/−^ mice exposed to both room air and cigarette smoke for 6 months did not display any significant urothelial damage. The number of breaks in the urothelium were quantified for each animal group (four animals in each group) and normalized to 1 for the wild‐type animals exposed to room air. As shown in the lower panel of Figure 6, wild‐type mice exposed to smoking for 6 months (black bars) had fivefold more breaks in the urothelium than in those animals exposed to room air (open bars). Minimal urothelial damage was observed in iPLA_2_ *β* ^−/−^ mice exposed to cigarette smoking or room air (Fig. 6, lower panel).

**Figure 7:**
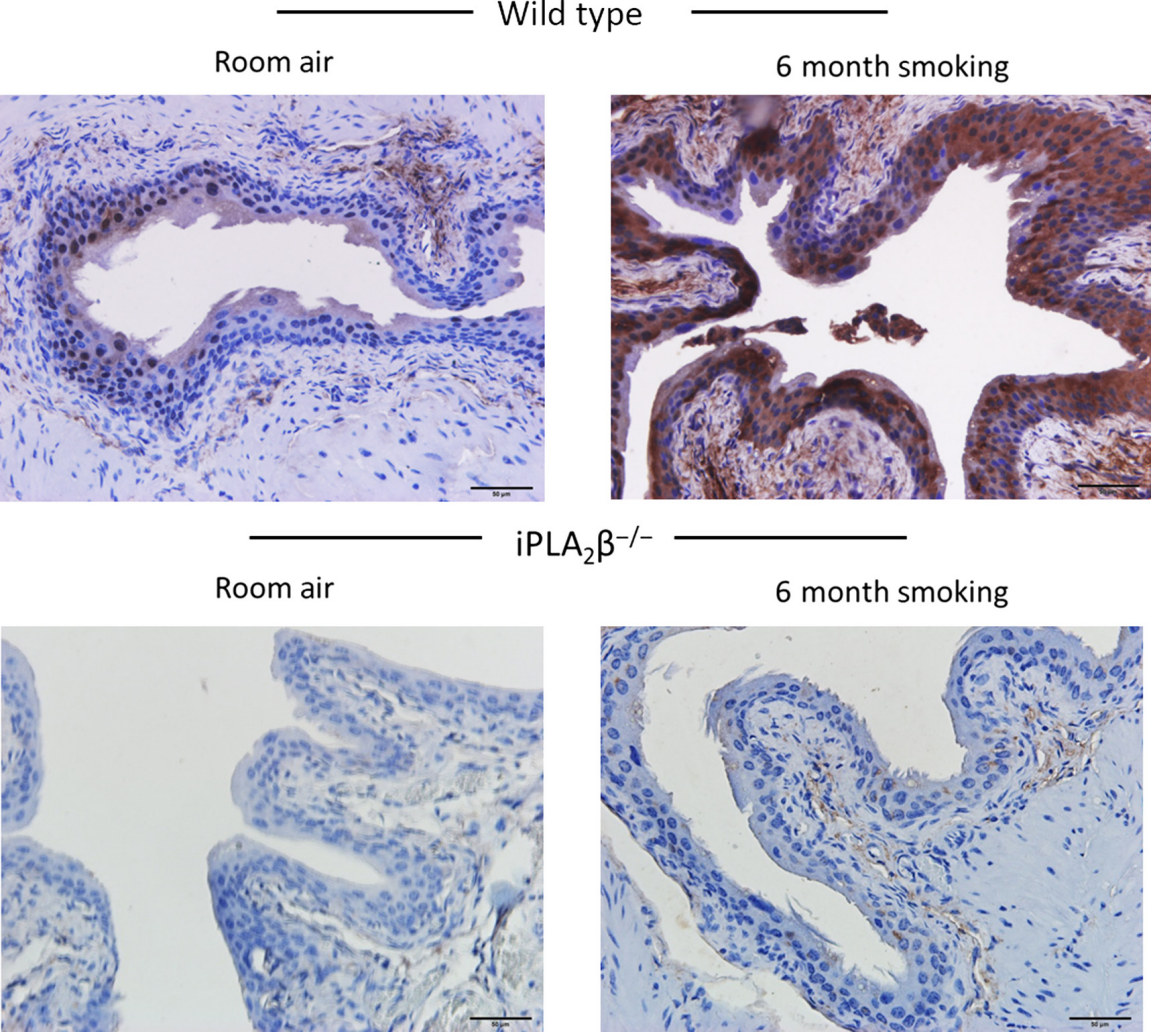
Representative immunohistochemistry staining of platelet‐activating factor (PAF) in mouse bladder sections. Top panels: Bladder sections from wild‐type female mice exposed to room air (left) or 6 months cigarette smoke (right). Bottom panels: Bladder sections from iPLA_2_ *β* ^−/−^ female mice exposed to room air (left) or 6 months cigarette smoke (right). Three animals in each group

**Figure 8:**
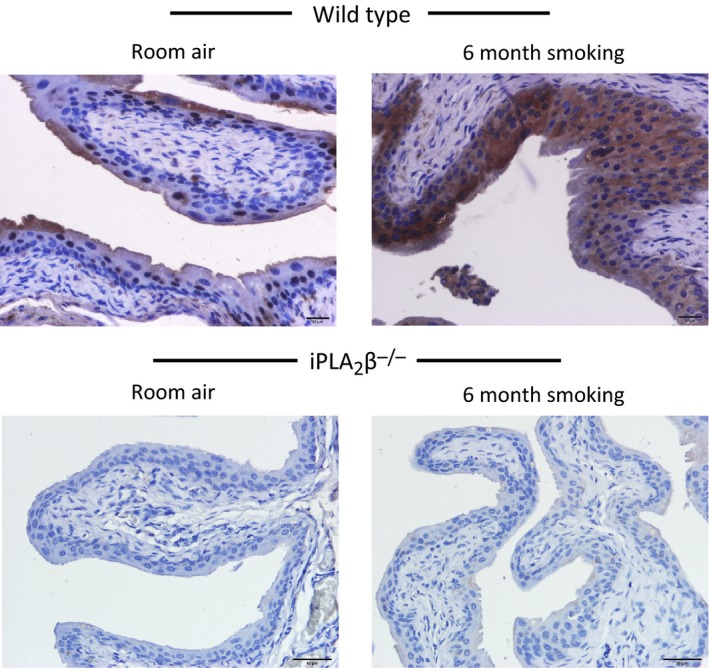
Representative immunohistochemistry staining of platelet‐activating factor (PAF) receptor in mouse bladder sections. Top panels: Bladder sections from wild‐type female mice exposed to room air (left) or 6 months cigarette smoke (right). Bottom panels: Bladder sections from iPLA_2_ *β* ^−/−^ female mice exposed to room air (left) or 6 months cigarette smoke (right). Three animals in each group.

Wild‐type mice exposed to cigarette smoking for 6 months showed increased PAF expression in the bladder urothelium when compared to mice exposed to room air (Fig. 7, top panels). Urothelial cell PAF expression was negligible in the bladders of iPLA_2_ *β*‐KO mice in the presence of room air or 6 months smoking (Fig. 7, bottom panels). The increased PAF expression in the bladders of WT mice in the presence of 6 months smoking was accompanied by a significant increase in urothelial PAF receptor expression (Fig. 8, top panels). PAF receptor expression was not detected in the bladder urothelium of iPLA_2_ *β* ^−/−^ mice exposed to room air or 6 months smoking (Fig. 8, bottom panels). Thus, long term cigarette smoking was associated with increased PAF and PAF receptor expression, accompanied by breaks in the urothelium. These changes were absent in the bladders of iPLA_2_ *β* ^−/−^ mice.

## Discussion

Despite being identified for over 100 years, the etiology, pathophysiology and treatment of IC/BPS remain elusive and incompletely understood. IC/BPS is associated with a number of risk factors, including gender, race and socioeconomic status. Additionally, modifiable risk factors as diet and smoking have been identified (Wu et al. 1993; Yan et al. 1993). To date, the mechanism underlying cigarette smoke‐induced bladder pain has not been elucidated. In a recent study (Marentette et al. 2015), we showed that cigarette smoking was associated with increased PAF production and adherence of inflammatory cells to the bladder endothelium. Additionally, mice exposed to cigarette smoking for 4 weeks showed increased accumulation of inflammatory cells in the bladder wall (Marentette et al. 2015).

Stimulus‐induced PAF production occurs via the remodeling pathway initiated by the activation of iPLA_2_ and accelerated hydrolysis of plasmenylethanolamine. The resultant lysoplasmenylethanolamine undergoes a transacylation reaction with plasmanylcholine to produce lyso‐PAF, which is in turn acetylated to form biologically active PAF. The biological activities of PAF can be rapidly terminated by PAF‐acetylhydrolases, a family of unique iPLA_2_ enzymes that hydrolyze the acetyl group at the *sn*‐2 position of PAF to generate biologically inactive lyso‐PAF and free acetate. This provides an immediate mechanism to prevent, control or terminate the proinflammatory effects elicited by PAF. Previous studies have shown that cigarette smoking is associated with inhibition of PAF acetylhydrolase, resulting in increased PAF content (Balsinde and Dennis 1996a; McHowat and Creer 1998; Sharma et al. 2010; Marentette et al. 2015). However, increased PAF in response to smoking can be inhibited by treatment with inhibitors of iPLA_2_ *β*, the predominant PLA_2_ isoform responsible for PAF production in endothelial (McHowat et al. 1993a, 1997, 2000; Cummings et al. 2000) and epithelial cells (Balsinde and Dennis 1996a). In this study, we show the expression of PAF and the PAF receptor is minimal in the bladder wall of mice exposed to room air, and is confined to the superficial layers of the urothelium. In contrast, staining for both PAF and the PAF receptor was intense and occurred throughout the entire urothelium in mice exposed to cigarette smoke. We show that PAF accumulation in urothelial cells exposed to CSE is blocked by pretreatment with the iPLA_2_ *β* inhibitor BEL (Fig. 2). Likewise, increased PAF accumulation was observed in the urothelium of wild‐type mice exposed to cigarette smoking but was absent in iPLA_2_ *β*‐/‐ mouse bladder (Fig. 7). The absence of iPLA_2_ *β* was also associated with preservation of the bladder urothelium in the presence of smoking (Fig. 6), suggesting that iPLA_2_ *β* mediates urothelial cell damage, possibly via PAF accumulation. It has been reported that patients with IC/BPS who are smokers experience symptom improvement upon smoking cessation. IC/BPS patients display both inflammation in the bladder wall as well as damage to the urothelium (Yamada et al. 1994; McHowat et al. 2000). In a previous study, we showed that smoking is associated with inflammatory cell recruitment into the bladder (McHowat et al. 1993a). Thus it is currently unclear as to whether inflammation caused by smoking damages the urothelium or if damage to the urothelium causes inflammation.

In Figure 1, we show that iPLA_2_ activity in immortalized urothelial cells isolated from IC/BPS patients is more susceptible to inhibition with (*S*)‐BEL than the activity measured in immortalized urothelial cells from non‐IC/BPS patients or human urothelial cells. Since (S)‐BEL is selective for the iPLA_2_ *β* isoform, these data indicate that iPLA_2_ *β* activity is greater in urothelial cells from IC/BPS patients and may be responsible for the increased PAF accumulation in IC/BPS cells as shown in Figure 3. The increase in iPLA_2_ *β* activity and PAF accumulation suggests that IC/BPS patients who smoke may be particularly susceptible to PAF‐mediated inflammatory responses in the bladder wall.

Our data in vitro and in vivo indicate that PAF accumulation was accompanied by increased PAF receptor expression. Once formed, PAF binds to its receptor to exert inflammatory responses. The signaling responses through PAF and its receptor include activation of matrix metalloproteinases (MMPs) (Fleer et al. 1981; Burke and Dennis 2009) that can ultimately disrupt cell‐cell junctions (Dennis et al. 2011) resulting in increased bladder permeability. In addition to inducing MMP expression, signaling through PAF and its receptor has been shown to induce expression of a number of inflammatory mediators such as cyclooxygenase‐2 (COX‐2), inducible nitric oxide synthase (iNOS) and nuclear factor kappa‐light‐chain‐enhancer of activated B cells (NF‐*κ*B) (Dan et al. 1996).

Urothelial cell integrity is essential for the maintenance of the barrier properties of the bladder. Disruptions in the urothelial barrier properties result in leakage of urine contents into the bladder wall, causing pain and inflammation, as observed in IC/BPS patients. To maintain the barrier of the urothelium, urothelial cells express tight junctional proteins, adherens junctions and secrete a glycosaminoglycan mucus layer that protect the bladder wall (Yamada et al. 1994; Sharma et al. 2011). Bladder biopsies of patients with IC/BPS have shown a reduced expression of tight junctional proteins and adherens junctions, rendering the urothelium permeable to urinary contents (Messner et al. 2008; Sharma et al. 2009). PAF accumulation and MMP activation in the bladder wall of patients who smoke may contribute to permeability of the urothelium via degradation of junctional proteins.

In this article we highlight a potential role of PAF and its receptor in the development and/or progression of IC/BPS. We observed that the urinary PAF content in IC/BPS patients was significantly higher than in control patients. This suggests that the urothelial cells of IC/BPS patients produce and release PAF, which supports the hypothesis that PAF is involved in IC/BPS. As PAF has been implicated in the breakdown of tight junctional protein expression (Xu et al. 2012) and promoting inflammation (Cummings et al. 2000; McHowat et al. 2001; Dennis et al. 2011) targeting of either the production of PAF through inhibition of iPLA_2_ *β* or blocking the PAF receptor could serve as a beneficial treatment option for managing IC/BPS. PAF receptor antagonism or iPLA_2_ *β* inhibition could potentially be used as an adjuvant therapy with current treatment options aimed at replenishing the lost urothelial barrier properties. PAF receptor antagonists have been developed and evaluated in several inflammatory conditions (Siebeck et al. 1994; da Silva et al. 1996; Baltas et al. 2003; Vogensen et al. 2003; Grypioti et al. 2008). *Ginkgo biloba* is a PAF receptor antagonist that is excreted unchanged in the urine (Braquet 1986; Becker et al. 1988) and may be particularly beneficial in the management of IC/BPS.

## Conclusion

In conclusion, we show that cigarette smoke exposure results in the increased production of PAF and increased expression of the PAF receptor in the bladder urothelium. We further show that the observed increase in PAF production is more substantial in urothelial cells derived from IC/BPS patients. Patients with IC/BPS display higher urinary PAF content compared to healthy patients and the PAF content is greater in IC/BPS patients who smoke. Our in vivo studies suggest that cigarette smoke‐induced PAF production may directly damage the urothelium, contributing to leakage of urinary contents into bladder wall of patients with IC/BPS, setting up a cycle of pain and inflammation.

## Conflict of Interest

None declared.
